# Design of a High-Performance Micro Integrated Surface Plasmon Resonance Sensor Based on Silicon-On-Insulator Rib Waveguide Array

**DOI:** 10.3390/s150717313

**Published:** 2015-07-16

**Authors:** Dengpeng Yuan, Ying Dong, Yujin Liu, Tianjian Li

**Affiliations:** Graduate School at Shenzhen, Tsinghua University, J209A, Tsinghua Campus, University Town of Shenzhen, Shenzhen 518055, China; E-Mails: ydp12@mails.tsinghua.edu.cn (D.Y.); liuyj13@mails.tsinghua.edu.cn (Y.L.); litj13@mails.tsinghua.edu.cn (T.L.)

**Keywords:** surface plasmon resonance (SPR), silicon-on-insulator (SOI), biochemical sensor, rib waveguide, finite-difference time-domain (FDTD) method

## Abstract

Based on silicon-on-insulator (SOI) rib waveguide with large cross-section, a micro integrated surface plasmon resonance (SPR) biochemical sensor platform is proposed. SPR is excited at the deeply etched facet of the bend waveguide by the guiding mode and a bimetallic configuration is employed. With the advantages of SOI rib waveguide and the silicon microfabrication technology, an array of the SPR sensors can be composed to implement wavelength interrogation of the sensors’ output signal, so the spectrometer or other bulky and expensive equipment are not necessary, which enables the SPR sensor to realize the miniaturization and integration of the entire sensing system. The performances of the SPR sensor element are verified by using the two-dimensional finite-different time-domain method. The parameters of the sensor element and the array are optimized for the achievement of high performance for biochemical sensing application. As a typical example, a single bimetallic SPR sensor with 3 nm Au over 32 nm Al possesses a high sensitivity of 3.968 × 10^4^ nm/RIU, a detection-accuracy of 14.7 μm^−1^. For a uniparted SPR sensor, it can achieve a detection limit of 5.04 × 10^−7^ RIU. With the relative power measurement accuracy of 0.01 dB, the refractive index variation of 1.14 × 10^−5^ RIU can be detected by the SPR sensor array.

## 1. Introduction

Surface plasmon resonance (SPR) phenomenon has been widely applied in biochemical sensing in recent decades, and this technology possesses a profound developing potential due to their unique advantages for real-time in-situ and label-free sensing [1,2]. The SPR-based sensors have exhibited high performance, especially the very small detection limit of physical and biochemical parameters, such as the refractive index of analyte solution, DNA, protein, and bacteria [3,4]. Lab-on-chip integration is the trend of SPR-based sensor for biochemical applications in the last decade [4], coming with many new principles, excitations, configurations and materials related to SPR [5].

The most common SPR sensors are based on Kretschmann prism configuration, which is bulky and not compatible with the current MEMS technology, and therefore not good for realizing miniaturized integrated sensing system [6]. As another popular configuration, the fiber optic SPR biosensor require complex micromachining process and some high-performance equipment, it is difficult to achieve low cost and small size of the overall sensing system [7,8]. The silicon-on-insulator (SOI) rib waveguide with large cross-section, by contrast, can achieve low-loss propagation and high integration with the optical fiber communication systems and the (opto-) electronic systems, so it is able to provide a competitive development platform for the SPR sensing applications. However, the requirement for the evanescent field of waveguide or fiber to excite the SPR phenomenon is that the propagation constant of the guiding mode must match the wave vector of SPR [9]. Obviously, for a SOI rib waveguide with large cross-section, the mode effective index is much larger than the refractive index of the general analyte solution, so this traditional SPR excitation is not feasible. In this paper, a new SPR excitation is proposed and analyzed.

In general, there are several metals can be used to excite the SPR, like gold (Au), aluminum (Al) or silver (Ag). As the most common SPR active metal, gold (Au) possesses better chemical stability and exhibits a larger shift in resonance wavelength than other metals, which leads to a very high sensitivity of SPR sensor [10]. However, due to its high absorption of light, the resonance curve of Au SPR sensor is broader and bad for the performance of the sensor [11]. By comparison, Al and Ag are known for their narrower spectral width of SPR curves, but they are easily oxidized when used in liquid or gaseous environments because of their poor chemical stability [12,13]. Moreover, Al is more economical than Au or Ag, and it can be deposited directly over the silicon layer so that other transitional material layers are not necessary. As compared to a single metallic layer, the bimetallic configuration with a few nanometers Au over Al make the SPR sensor exhibits better performance and effectively protects Al from oxidation [11,14]. In this article, the sensor performance with respect to Al and Au will be specifically analyzed and the bimetallic configuration will be employed.

Usually, angular and wavelength interrogation are the most prevalent detection approaches for the biochemical sensors based on SPR, so the high performance of sensors is guaranteed by the high resolution tunable laser sources and optical spectrometers or high-precision angle sweeping equipment. These external attachments are expensive and cumbersome; the whole measurement systems are not low cost and difficult to integrate, so an overwhelming majority of SPR sensors are confined in laboratories. With the advantages of the silicon microfabrication technology, a SOI rib waveguide array can be realized easily. Using an array of light sources with a sequence of wavelengths for the SPR sensor array, wavelength interrogation can be implemented and the resonance wavelength of SPR can be determined from the response of the photodetector array rather than the scanning and measurement of wavelength, therefore no spectrometer is needed. Similarly, angular interrogation can also be implemented by a suite of array elements with different angles. Making full use of sensor array is not only conducive to integration and package of the whole sensing system but also reduces the cost of production.

As the requirements of the biochemical *in-situ* real-time sensing increase, the research of miniaturized and integrated high-performance SPR biochemical sensors has become necessary. This article proposes a micro integrated surface plasmon resonance (SPR) biochemical sensor platform based on silicon-on-insulator (SOI) rib waveguide with large cross-section. Following the introduction to the background of the research, the principles of operation are presented in Section 2, including the structure configuration of SPR sensor, calculation model and three quantitative indicators of sensor performance. In Section 3, the results of the investigation are analyzed and discussed. The conclusions of the research will be given in the last section.

## 2. Principle of Operation

### 2.1. Structure Configuration

**Figure 1:**
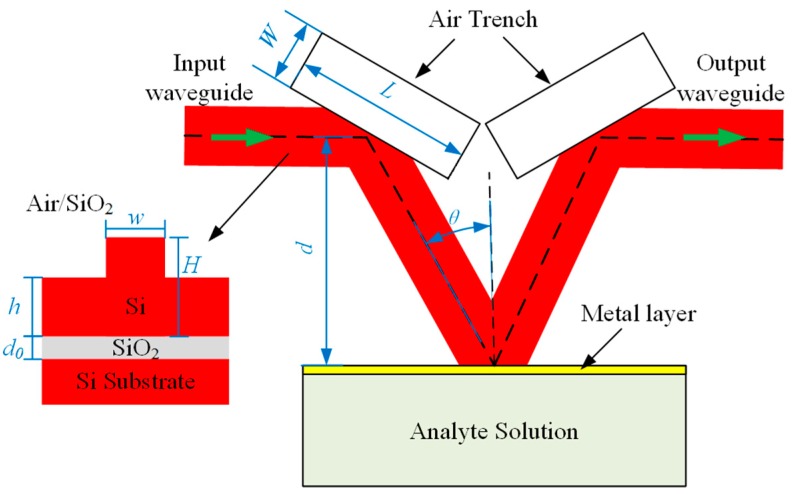
The top view of the waveguide based surface plasmon resonance (SPR) sensor and the schematic cross-section of the silicon-on-insulator (SOI) rib waveguide embedded in the lower left corner.

The proposed SPR sensor based on SOI rib waveguide with large cross-section is shown in Figure 1. It is comprised of an input waveguide, an output waveguide, a V-shaped bend waveguide, two air trenches and a deeply etched facet coating with metal layer as the SPR interface. In this work, the analyte solution is supposed to be water and the refractive index is in the vicinity of 1.33. The two deeply etched air trenches are used to connect the V-shaped waveguide with the input waveguide and the output waveguide, therefore two bends of the related rib waveguides are formed. Thus the input and output waveguides are in a straight line and parallel to the SPR interface. The distance between the SPR interface and the centerline of the input and output waveguides is *d*, the half-angle of the V-shaped bend waveguide is θ, and *W* and *L* represent the width and length of the two air trenches, respectively.

The image embedded in the lower left corner of Figure 1 is the schematic cross-section of the employed SOI rib waveguide, where *H*, *h*, *w*, and *d_0_* are geometries. The upper cladding material of SOI rib waveguide is air or silicon dioxide (SiO_2_) generally, and it is assumed to be air in this paper. To obtain high coupling efficiency of the SOI rib waveguide with the standard single-mode glass fibers, the total rib height (*H*) is set to 10 microns, and the input and output waveguides can easily connect to the laser light sources and the external spectrometer or optical power meters.

**Figure 2:**
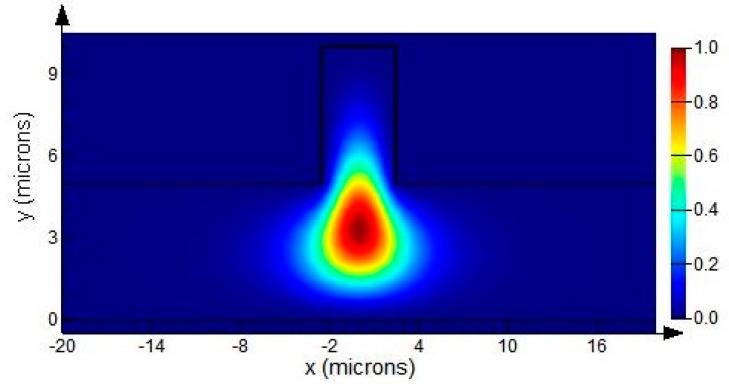
The mode distribution of the SOI rib waveguide with *H =* 10 μm, *h =* 5 μm and *w =* 5 μm at an operating wavelength of 1550 nm.

Generally, in order to achieve low-loss propagation and avoid the negative influences due to multimode transmission, the optical waveguides employed in the sensor must possess single-mode. Soref, Rickman, Powell *et al.* have proposed and studied the single-mode conditions of SOI rib waveguide with large cross-section [15,16,17,18], which can be expressed as:

(1) r≥0.5

(2) $$t<c+\frac{r}{\sqrt{1-r^{2}}}$$

where *r = h/H*, *t = w/H*, and *c* is a constant. Equations (1) and (2), respectively, represent the single-mode propagation condition for SOI rib waveguide with large cross-section in the vertical and lateral directions. When these two conditions are satisfied simultaneously, the SOI rib waveguide with large cross-section can be considered as “single-mode”. Employing a strict single-mode condition [18], the SOI rib waveguide with a total rib height of 10 microns is set to possess an outside rib height (*h*) of five microns, rib width (*w*) of five microns, and thickness of SiO_2_ insulator layer (*d_0_*) of two microns, thus only the fundamental guiding modes for each polarization are able to survive. In addition, the guiding mode of the SOI rib waveguide must be transverse electric (TE) polarization because of the polarization selectivity of SPR excitation. At a wavelength of 1550 nm and the corresponding refractive indices (*n_Si_ =* 3.476, *n_SiO2_ =* 1.444, *n_Air_ =* 1), the fundamental TE polarization mode distribution can be calculated by using a finite difference method (FDM) [19] with the perfectly-matched layers (PMLs) boundary treatment [20], as shown in Figure 2.

Obviously, it is difficult to adopt angular interrogation for one uniparted SPR sensor because the parameter θ is a constant after the structure processing is completed, but wavelength interrogation is available. The range of the operation wavelength was chosen to be from 1200 nm to 1800 nm where the SOI material is transparent and the standard optical fiber can be used for interconnection. In the operation, the light is coupled into the input waveguide and propagates along the SOI rib waveguides with large cross-section. At the appropriate operating wavelength under the specified parameters, the SPR phenomenon can be excited at the metal–analyte interface, and the light intensity detected from the output waveguide will exhibit minimum. In the entire propagation process, the guiding modes of the rib waveguides will be reflected three times. In order to ensure a sufficiently high reflection efficiency of the two bends of the input and output waveguides, the parameters *W* and *L* must be large enough, there setting *W =* 5 μm, *L =* 20 μm. Moreover, considering the mode spot size of the SOI rib waveguide shown in Figure 2, we set *d =* 25 μm, which is large enough to prevent the input and output waveguides interfering the excitation of SPR at the metal-analyte interface.

### 2.2. Calculation Model

**Figure 3:**
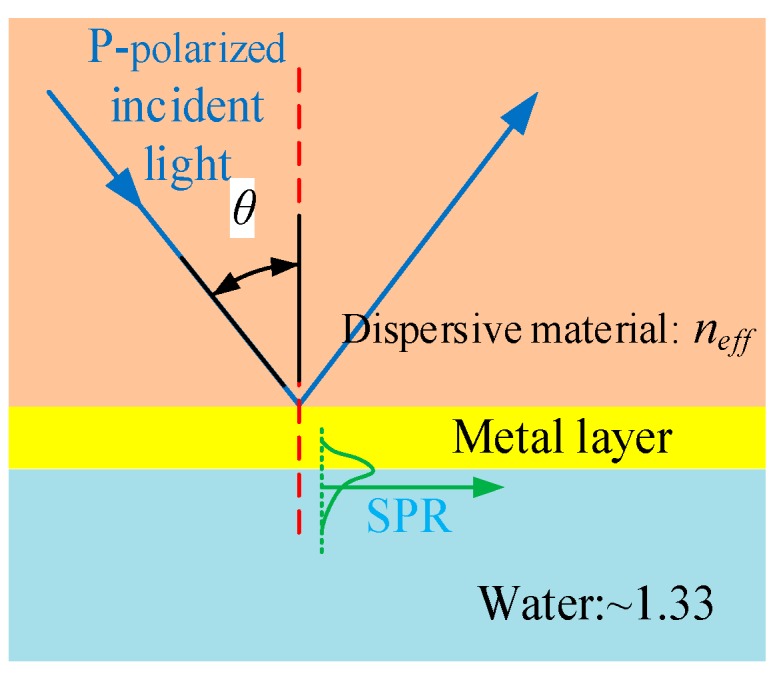
The simplified SPR excitation model of the SPR sensor.

According to Figure 1, the two dimensional (2D) SPR sensor can be numerically modeled and simulated by using 2D finite-difference time-domain (FDTD) method [21] with PMLs. Based on the effective index method (EIM) [22], the SOI rib waveguide structure was approximated as a 2D structure for these calculations. It is well known that the higher accuracy of FDTD simulation, the more time consumption and memory requirements of computers. With approximately 5 h needed for per simulation on an *Intel core i7 processor* with the CPU Clock Speed of 3.3 GHz, it is impossible to employing the 2D-FDTD to simulate the SPR sensor and analysis its performance, which is based on a large amount of data. Essentially, the SPR excitation mechanism of this proposed SPR sensor based on SOI rib waveguide is analogous to the classical SPR model by using a prism in the Kretschmann geometry [23], but the resonance condition must be improved and can be expressed as Equation (1).

(3) $$k_{0}n_{eff}\sin\theta =K_{SPR}=k_{0}\sqrt{\frac{\varepsilon_{m}\varepsilon_{d}}{\varepsilon_{m}+\varepsilon_{d}}}$$

where, *K_SPR_* is the SPR propagation constant at the metal–analyte interface, *n_eff_* is the effective refractive index of the guiding mode of the SOI rib waveguide; ε*_m_* and ε*_d_* are the dielectric constants of the metal and the analyte solution, respectively. *k_0_ =* 2π/λ_0_, and λ*_0_* is the free space optical wavelength. Therefore, this SPR sensor can be simplified as a special Kretschmann model, including a virtual prism whose refractive index is equal to the effective refractive index of the guiding mode and a p-polarized incident beam evolved from the TE-polarized guiding mode of the SOI rib waveguide, as shown in Figure 3. So the SPR curve and the resonance parameters of this SPR sensor can be calculated rapidly using N-layer transfer matrix method [24].

To reduce time consumption and complexity, this analytical method was used to design parameters and analyze the performance of the proposed SPR sensor, and the 2D-FDTD simulation with PMLs was employed to obtain the optical transmission distribution and verify the results.

### 2.3. Dispersive Material Model

In this paper, we use the Drude model for the dispersion of SPR metal [25], given as

(4) $$\varepsilon_{m}(\lambda )=1-\frac{\lambda^{2}\lambda_{c}}{\lambda_{p}^{2}(\lambda_{c}+i\lambda )}$$

where, λ*_p_* and λ*_c_* denote the plasma wavelength and collision wavelength, respectively. For gold (Au), λ*_p_ =* 168.26 nm, λ*_c_ =* 8934.2 nm; and for aluminum (Al), λ*_p_ =* 106.57 nm, λ*_c_ =* 24,511 nm [26].

**Figure 4:**
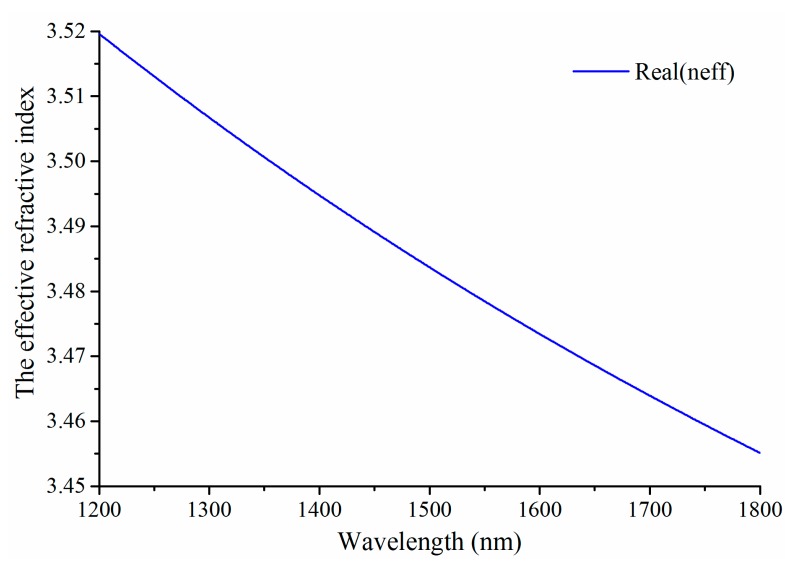
The dispersion of the fundamental transverse electric (TE) guiding mode of the SOI rib waveguide with the total rib height of 10 μm, outside rib height of 5 μm, rib width of 5 μm.

The dispersion of the fundamental TE-polarized guiding mode of the SOI rib waveguide (as shown in Figure 2) can be obtain by using a FDM with PMLs, and Si and SiO_2_ in SOI also employ the dispersive data [27]. The curve about the effective refractive index *n_eff_* with respect to the wavelength of incident light is shown in Figure 4.

### 2.4. Quantitative Indicators of Sensor Performance

For quantifying the sensor performance, the SPR sensor performance is determined in terms of two characteristics generally, including sensitivity (*S_n_*) and detection-accuracy (*DA*) [14]. For wavelength interrogation, the sensitivity is defined as the ratio of the shift in resonance wavelength (Δλ*_Res_*) for a given change (Δ*n_a_*) of the analyte solution, *i.e*., *S_n_ =* Δλ*_Res_/*Δ*n_a_*. The detection-accuracy determines the accuracy of measurement of resonance wavelength and is defined as the reciprocal of full-width-at-half-minimum (*FWHM*) of the SPR curve, *i.e.*, *DA = 1/FWHM*. Similarly, the sensitivity and detection-accuracy of angular interrogation can be also defined. Moreover, *Contrast = (R_max_ − R_min_)/(R_max_ + R_min_)* is defined to indicate the contrast of the output signal, where *R_min_* and *R_max_* represent the normalized output light intensity at the resonance point and far from the resonance point of the SPR curve. For high-performance SPR sensors, the sensitivity, detection-accuracy and *Contrast* should be as large as possible.

## 3. Results and Discussion

### 3.1. Single Metallic SPR

**Figure 5:**
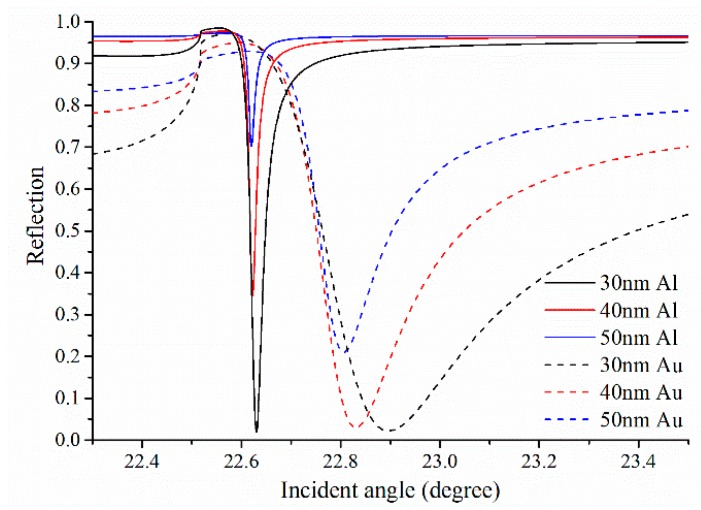
The single metallic SPR curve of angular interrogation for different thickness of the metal (Au or Al) layer at operating wavelength of 1550 nm and the refractive index of the analyte solution *n_a_ =* 1.33.

**Figure 6:**
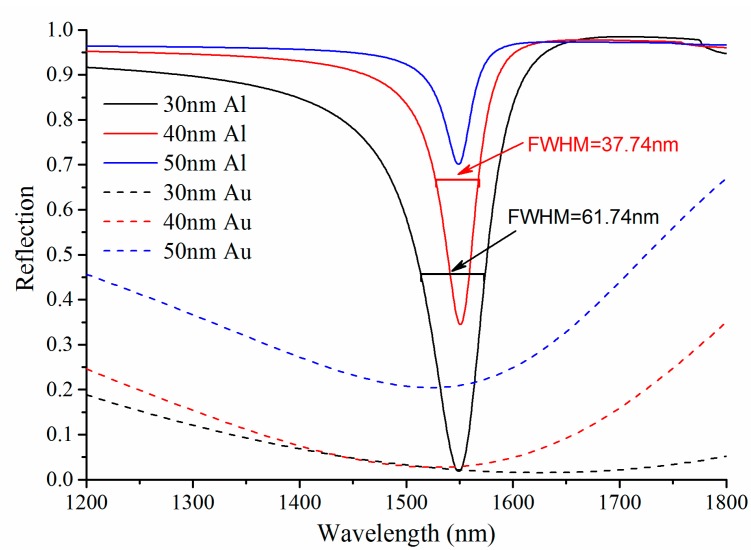
The single metallic SPR curve of wavelength interrogation for different thickness of the metal (Au or Al) layer at resonance angle from Figure 5 and the refractive index of the analyte solution *n_a_ =* 1.33.

The single metallic SPR sensors used Au or Al are analyzed and compared, Figure 5 and Figure 6 are the SPR curve of angular interrogation and wavelength interrogation, respectively. To ensure the resonance wavelengths within the specified spectral range (1200~1800 nm) with a central wavelength of 1550 nm, the SPR curves of wavelength interrogation employ the resonance angles from Figure 5 as the light incident angle θ. It is found that the changes of the metal-layer thickness lead to the variation of the resonance positions, and the reason is that the propagation constant of multilayer SPR with a thin metal layer is different to that of ideal semi-infinite configuration as the *K_SPR_* expressed in Equation (1). Due to a larger absorption coefficient, Au SPR exhibits broader resonance curve, which leads to a larger *FWHM* (lower *DA*) and even out of the specified spectral range. For the same reason, the *Contrast* of Al SPR curve is lower than that of Au SPR with the same thickness of the metal-layer. Furthermore, the Al SPR sensors with thinner metal layer exhibit higher *Contrast* and larger *FWHM* (lower *DA*), so a tradeoff of the Al-layer thickness is necessary for high performance of Al SPR sensors. For example, the detection-accuracy of 30 nm and 40 nm Al SPR sensor are 16.2 μm^−1^ and 26.5 μm^−1^, respectively.

**Figure 7:**
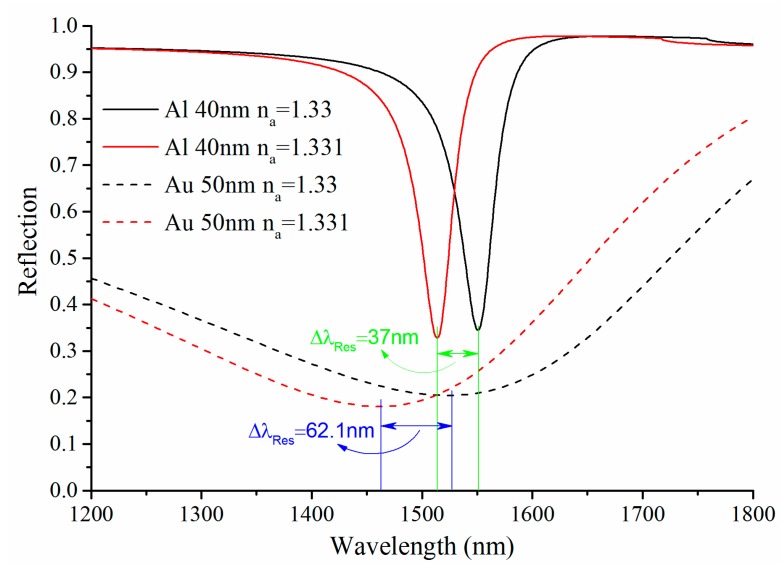
The single metallic SPR curve of wavelength interrogation for different SPR active metal (Au or Al) and different refractive index of the analyte solution.

For 40 nm Al and 50 nm Au SPR sensors, the shifts in resonance wavelength (Δλ*_Res_*) for a given change Δ*n_a_ =* 0.001 of the refractive index of the analyte solution are shown in Figure 7. Al SPR exhibits smaller shift as compared to Au, so the sensitivity of Al SPR sensor is lower than that of Au SPR sensor. There, the sensitivity of 40 nm Al and 50 nm Au SPR sensor are 3.7 × 10^4^ nm/RIU and 6.21 × 10^4^ nm/RIU, respectively. Despite all this, the overall performance of the single metallic SPR sensor based on Al is better than Au, especially for this SOI rib waveguide platform with operation wavelength within the specified spectral range from 1200 nm to 1800 nm.

In addition, the proposed SPR sensor is based on SOI material, it is difficult to directly deposit the Au film on the etched facet of the silicon layer. The common approach is to pretreat the surface by depositing a titanium film, which will undoubtedly increases the complexity of processing and leads to greater error. Compared to Au, Al is fully compatible with SOI material and relatively economical, which significantly reduces the cost of the sensor. Under the comprehensive consideration, the Al SPR sensors have better performance even though sacrificing a fraction of sensitivity.

### 3.2. Bimetallic SPR

**Figure 8:**
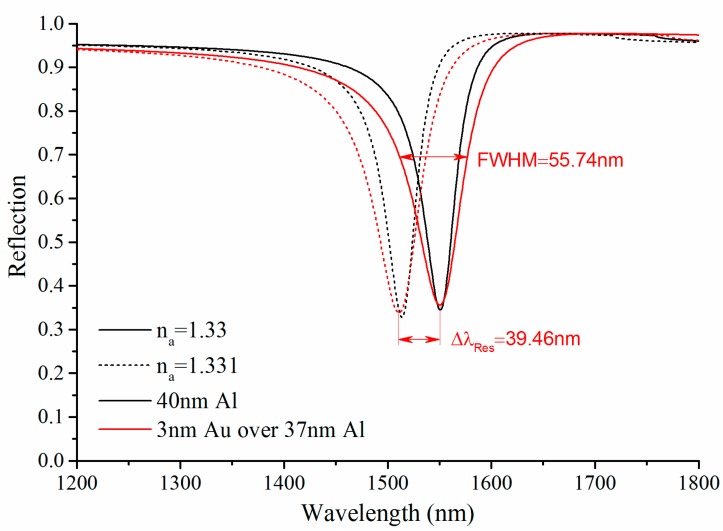
The comparison diagram of wavelength interrogation curve for single metallic SPR with 40 nm Al and bimetallic SPR curve with 3 nm Au over 37 nm Al.

Al is easily oxidized because its poor chemical stability and its oxidation will influence the performance of Al SPR sensors [12,13]. Making use of the bimetallic configuration (coating an ultra-thin layer of Au over Al) is a good solution to avoid this problem [11,12,14]. Supposing 3 nm Au deposited over 37 nm Al, the bimetallic SPR curve is shown in Figure 8. In this case, the sensitivity and *FWHM* of bimetallic SPR sensor is larger slightly than that of single metallic SPR sensor with the same thickness of metal layer, but the *Contrast* is almost unchanged. It is concluded that the bimetallic SPR sensor with 3 nm Au over 37 nm Al inherits the advantages of the single metallic SPR sensor with 40 nm Al, and the several nanometers Au can protect Al from oxidization.

**Figure 9:**
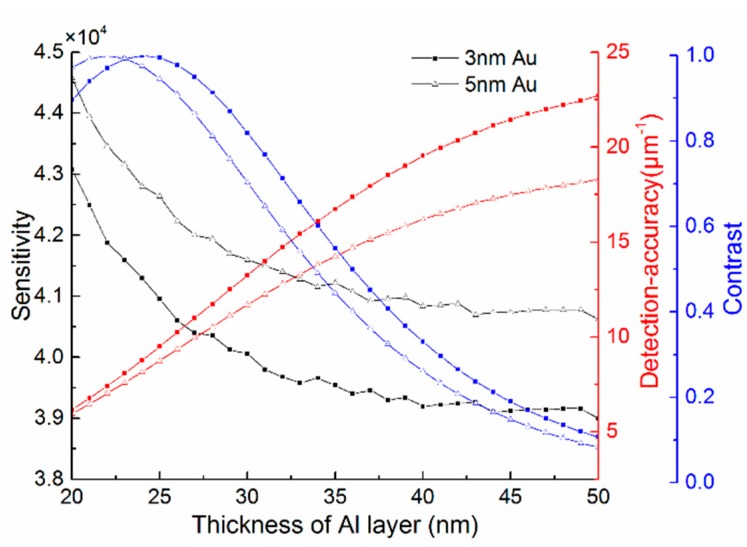
The influence of the thickness of Al layer in the bimetallic SPR sensor with 3 nm or 5 nm Au.

**Figure 10:**
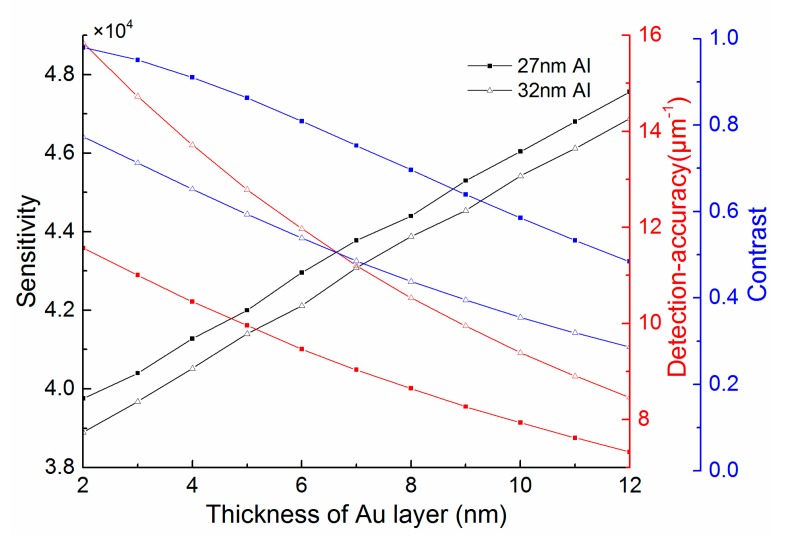
The influence of the thickness of Au layer in the bimetallic SPR sensor with 27 nm or 32 nm Al.

For the bimetallic configuration with a few nanometers Au layer deposited over Al, the thicknesses of each metal layer have influence on the performance of the SPR sensor. It can be seen from Figure 9 that the bimetallic SPR sensor exhibits highest sensitivity *S_n_ =* 4.458 × 10^4^ nm/RIU with 20 nm Al and 5 nm Au, but smallest *DA =* 5.95 μm^−1^. In addition, the *Contrast* achieves a maximum in thinner Al layer (about 22 nm) and has an overall downtrend. The thinner Al layer leads to higher sensitivity and *Contrast* but lower *DA* of SPR sensor, so there is a tradeoff between the sensitivity, *Contrast* and *DA*. On the other hand, with the thickness of Au increasing, the sensitivity of SPR sensor increases linearly, but the *DA* and *Contrast* become worse, as shown in Figure 10. It is worth noting that the influence of Au layer thickness is smaller than that of Al, therefore, a few nanometer Au layer retains the sensor’s high performance along with protects Al from oxidation.

After comprehensive consideration, there is a trade-off decision that the limited thickness ranges of metal layer are from 25 nm to 35 nm for Al and from 2 nm to 5 nm for Au. As a typical example, the bimetallic SPR sensor with 3 nm Au over 32 nm Al possess a high sensitivity of 3.968 × 10^4^ nm/RIU, a detection-accuracy of 14.7 μm^−1^ and a *Contrast* of 0.82. Supposing the resolution of the spectrometer connected to the output waveguide is 0.02 nm, the bimetallic SPR based on SOI rib waveguide can achieve a refractive index detection limit of 5.04 × 10^−7^ RIU. The achieved detection limit is lower than that obtained by the sensor based on Young’s interference (9 × 10^−9^ RIU) [28], but is in the same order as the counterparts (10^−5^–10^−7^ *RIU*) that reviewed in reference [3,4].

### 3.3. Transmission Simulation of SPR Sensor

**Figure 11:**
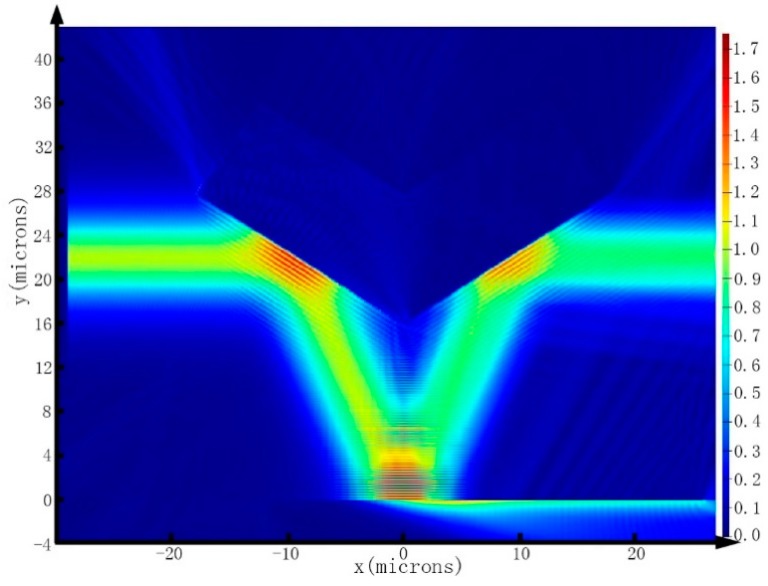
The electric intensity map of the bimetallic SPR sensor with 3 nm Au over 32 nm Al in the 2D-FDTD simulation at an operating wavelength of 1550 nm. The mesh grid in the regions of trench-based waveguide bends is set to 5 nm, and the mesh grid of metal layer is set to 1 nm.

According to the structure configuration shown in Figure 1, and employing the resonance parameters from above analytical method, the bimetallic SPR sensor with 3 nm Au over 32 nm Al is simulated by using the 2D-FDTD with PMLs, and its optical transmission distribution is shown in Figure 11. Here, the TE-polarized fundamental mode of the SOI rib waveguide acted as the source incident into the input waveguide, and the half-angle of the V-shaped bend waveguide θ = 22.65*°*, which was calculated by the above analysis method with the effective model at resonance wavelength of 1550 nm.

It can be seen obviously that the SPR phenomenon was excited by the guiding mode at the metal-analyte interface. Unfortunately, only about 25 percent of the light energy was absorbed by SPR, which is not consistent with the *Contrast =* 0.82 that calculated from above analytical method. There are several aspects that lead to this situation. Firstly, because of the simplified effective model, the analytical method cannot take the spatial angular distribution of the mode source into account, which is the main reason for the large difference of *Contrast*. Secondly, the FDTD method itself is a discrete numerical solver for Maxwell equations, and the EIM is employed to realize 2D-FDTD to simulate propagation of the waveguide mode and excite SPR phenomenon, which undoubtedly will bring some errors. Thirdly, the dispersion model has some errors inevitably, especially the effective refractive index of the guiding mode. In addition, these above reasons lead to a certain errors of SPR resonance position, which will also cause the deviation of the signal contrast.

Even so, we can draw a conclusion exactly that the SPR phenomenon can be excited at the waveguide bends by the guiding mode, and the resonance parameters from the N-layer transfer matrix method are consistent with the 2D-FDTD simulation methods within a reasonable range of error. Therefore, the foregoing analysis using the analytical method is reliable.

### 3.4. SPR Sensor Arrays

**Figure 12:**
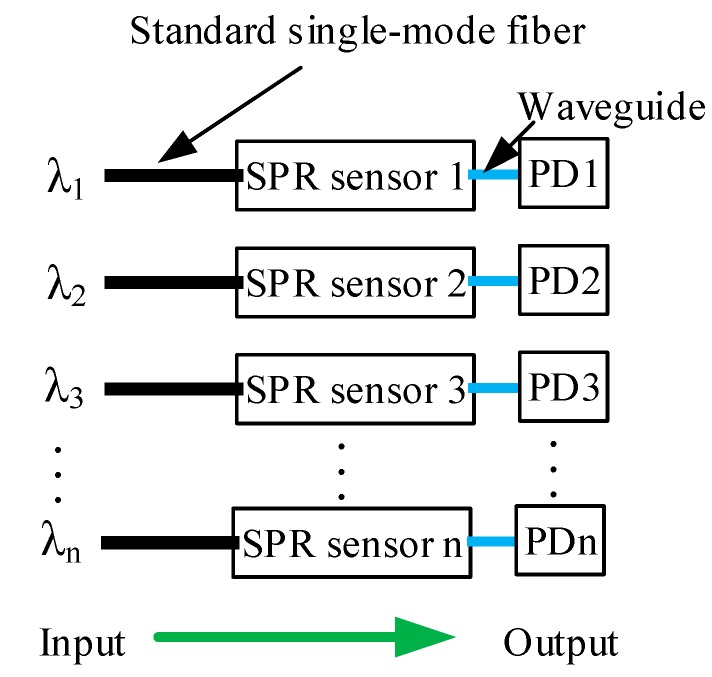
Schematic of SPR sensor array for wavelength interrogation.

Based on silicon microfabrication technology, an array can be used to implement the wavelength interrogation, as shown in Figure 12. Light with different wavelength from each element of the source array is coupled into the input waveguide of each element of the SPR sensor array through a standard single-mode fiber. A photodetector array is integrated at the end of the output waveguides and detects the output signal. For a set of operating wavelengths, the wavelength response of the SPR sensors can be obtained from the photodetector array, and the resonance wavelength of SPR can be determined, so the parameters of the analyte solution can be estimated.

**Figure 13:**
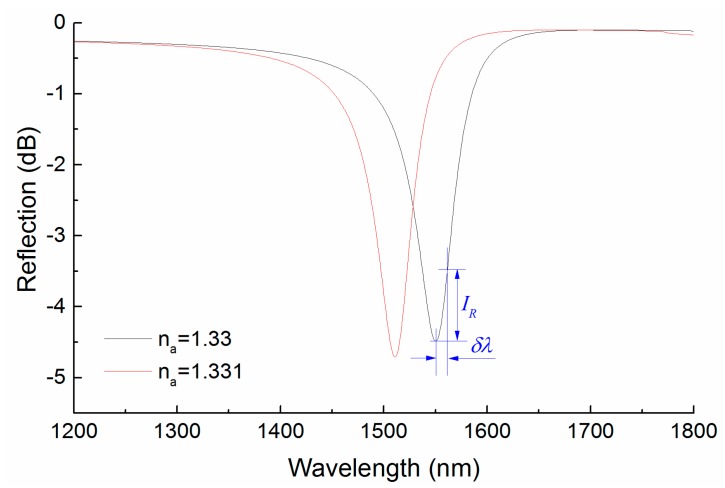
The wavelength interrogation curve for the bimetallic SPR curve with 3 nm Au over 32 nm Al.

As the output signal is produced by the photodetector array and the operating wavelength is discrete, the refractive index based detection limit of this SPR sensor array is determined by the wavelength resolution (δλ) of the adjacent sensor element, which is closely related to the relative power measurement accuracy (*IR*) of the photodetector [29]. The wavelength resolution (δλ) of adjacent sensor element can be obtain from the SPR resonance curve of almost continuous wavelength interrogations, using the N-layer transfer matrix method with a very small calculating increment of wavelength, as shown in Figure 13. Generally, the relative power measurement accuracy can reach a valve of 0.01 dB, the wavelength resolution of adjacent sensor element of SPR sensor array with 3 nm Au over 32 nm Al can be found, δλ = 0.44 nm. With the high sensitivity of 3.968 × 10^4^ nm/RIU, the refractive index variation of 1.14 × 10^−5^ RIU can be detected.

Similarly, the angular interrogation can also be implemented by a suite of array elements with different angles. Using the same calculating method with wavelength interrogation, the sensitivity of angular interrogation for a uniparted bimetallic SPR sensor with 3 nm Au over 32 nm Al is 19 degree/RIU. Considering the difficulty of processing, the minimum angle difference between the adjacent SPR sensor elements is assumed to be 0.001*°*, thus the refractive index detection limit of the SPR sensor angular array is 5.3 × 10^−5^ RIU. Although this detection limit value is much lower than that obtainable by the single SPR sensor (5.04 × 10^−7^ RIU), the optical detection unit at the output port is the low-cost and integrated photodetector array instead of the expensive optical spectrum analyzer. In addition, some numerical fitting algorithms can be used to determine the resonant wavelength or the resonant angle accurately, such as the polynomial fitting, so the accuracy of the sensor can be more accuracy.

### 3.5. Discussion

The proposed SPR sensor is based on the SOI rib waveguide with large cross-section, and the SPR phenomenon is excited at the waveguide bend by the guiding mode. According to the above analyses and simulations, it is found that the proposed SPR sensor have the ability to perform bulk sensing with high sensitivity, which can be used to detect the concentration changes of the analyte solution, acting as a chemical sensor. If some suitable receptor molecules are immobilized at the surface of metallic layer, this SPR sensor can also be used to detect biological reactions with surface sensing.

Compared with conventional SPR sensors, the biggest difference is that the configuration structure of this proposed structure SPR sensor is very unique, so the processing quality may affect the actual performance of the sensor. At first, the angular V-shaped structure leads to the waveguide is slant and the guiding mode of SOI rib waveguide will be reflected three times, so there is a high demand for the processing of waveguide and the trenches. Secondly, because the SPR interface locates on a deeply etched facet at the end wall of the V-shaped waveguide bend, it is difficult to homogeneously deposit the metallic layer. Moreover, as the metallic layer structure is very thin, especially the several nanometers gold film, the surface roughness may not meet the requirements of the effective excitation of the SPR. In practice, we can etch the rib waveguides at SOI wafer with ordinary lithography processes, then cleave the sensor units to make the deeply etched facet of the V-shape waveguide bending to be able to deposit the metal layers by the vacuum evaporate plating technology. At present, these processes are being implemented by our research group.

For the arrays of SPR sensors, the consistency and the angular precision of each element also affect the performance of SPR sensors. Therefore, a higher precision processing quality is needed to get a higher sensor performance, and the processing costs will be more. In addition, the package of the overall sensing system will introduce some errors, such as the coupling loss of the standard single-mode optical fibers with the input waveguides, output waveguides, laser light sources and spectrometers. Similarly, the integrated effects of the photodetector array with the output waveguide of the array element cannot be ignored.

The proposed SPR sensor not only exhibits high sensitivity and detection-accuracy, but also benefits to the micro-integration, which provides a new possibility for the SPR sensor to commercial applications. Due to high coupling efficiency of the large cross-section SOI rib waveguide with standard single-mode glass optical fiber, the proposed SPR sensor can be conveniently integrated with optical fiber communication systems and (opto-) electronic systems. For a uniparted SPR sensor, the input and output waveguide can be remotely connected with a high-resolution tunable laser source and an infrared spectrometer through communication optical fibers, therefore the proposed SPR sensor has the potential to realize remote sensing, *in-situ* real-time detecting and possible application in internet of things. In addition, making use of the advantage of the silicon microfabrication technology, the SPR sensor array with discrete operating wavelengths is used to implement wavelength interrogation without the expensive and bulky spectrometer, and it can also be realized to detect some biochemical reactions. In a word, this proposed SPR sensor is theoretically analyzed and simulated in this paper, but the further experimental research is urgently needed, and this is the next task of our research team.

## 4. Conclusions

In this paper, a micro integrated SPR biochemical sensor platform based on SOI rib waveguide array and bimetallic SPR configuration (coating an ultra-thin layer of Au over Al) is proposed. The 2D-FDTD method and the N-layer transfer matrix method have been employed to analyze and simulate the performance of the proposed SPR sensor, and the optimized SPR sensors exhibit high performance. As a typical example, a single bimetallic SPR sensor with 3 nm Au over 32 nm Al possesses a high sensitivity of 3.968 × 10^4^ nm/RIU, and a refractive index detection limit of 5.04 × 10^−7^ RIU. Using the SPR sensor array to implement wavelength interrogation, the refractive index variation of 1.14 × 10^−5^ RIU can be detected.
